# The role of sirtuins in dermal fibroblast function

**DOI:** 10.3389/fmed.2023.1021908

**Published:** 2023-03-13

**Authors:** Michael M. Gilbert, Samuel C. Mathes, Avinash S. Mahajan, Craig A. Rohan, Jeffrey B. Travers, Anita Thyagarajan

**Affiliations:** ^1^Departments of Pharmacology and Toxicology, Boonshoft School of Medicine at Wright State University, Dayton, OH, United States; ^2^Departments of Dermatology, Boonshoft School of Medicine at Wright State University, Dayton, OH, United States; ^3^Edison Biotechnology Institute, Athens, OH, United States; ^4^Dayton Veterans Administration Medical Center, Dayton, OH, United States

**Keywords:** sirtuins, dermal fibroblast, senescence, aging, oxidative stress, wound healing

## Abstract

The sirtuins are a family of seven proteins that perform a variety of dermatological functions and help maintain both the structure and function of the skin. More specifically, the sirtuins have been shown to be altered in multiple dermal cell types including dermal fibroblasts. The functions of dermal fibroblasts are extensive, and include playing a significant role in wound healing as well as helping to maintain the integrity of the skin. As dermal fibroblasts age, they can undergo a state of permanent cell cycle arrest, known as cellular senescence. This senescent process can occur as a result of various stressors, including oxidative stress, ultraviolet radiation -induced stress, and replicative stress. In recent years, there has been a growing interest in both enhancing the cutaneous fibroblast’s ability to facilitate wound healing and altering fibroblast cellular senescence. Thus, in this review, we examine the relationship between sirtuin signaling and dermal fibroblasts to understand how this family of proteins may modulate skin conditions ranging from the wound healing process to photocarcinogenesis associated with fibroblast senescence. Additionally, we offer supporting data from experiments examining the relationship between fibroblast senescence and sirtuin levels in an oxidative stress model indicating that senescent dermal fibroblasts exhibit diminished sirtuin levels. Furthermore, we survey the research on the role of sirtuins in specific dermatological disease states that where dermal fibroblast function has been implicated. Finally, we conclude with outlining potential clinical applications of sirtuins in dermatology. In sum, we find that the literature on the involvement of sirtuins in dermal fibroblasts is limited, with research still in its early stages. Nevertheless, intriguing preliminary findings merit additional investigation into the clinical implications of sirtuins in dermatology.

## 1. Introduction

Sirtuins are nicotinamide adenine dinucleotide (NAD)^+^-dependent class III histone deacetylases (HDACs) that have been largely conserved throughout the evolutionary process. From prokaryotes to eukaryotes, their function has been studied in a variety of living species since the first sirtuin, Sir2 (silencing information regulator 2), was discovered in the yeast *Saccharomyces cerevisiae* (1–4). In mammals, seven sirtuins (SIRT1-7) have been discovered, each with a conserved catalytic core but different terminal domains (5, 6). Sirtuins are best known for their NAD^+^-dependent HDAC activity and also have a variety of additional functions depending in part on their cellular compartment (7). SIRT1 and SIRT6 have been extensively investigated and are primarily present in the nucleus, whereas SIRT2 can traverse the cytoplasm and enter the nucleus (8–11). The mitochondrial sirtuins, SIRT3, SIRT4, and SIRT5, have many roles in cellular homeostasis (12–14). Finally, SIRT7 can be found in the nucleolus and is less studied compared to the other sirtuins (11). Functionally, the sirtuins play multifaceted roles *via* epigenetic regulation in DNA damage repair, oxidative stress, cell cycle arrest, mitochondrial function, and telomere maintenance (14). As a result of their various functions, sirtuins are critical players in the homeostasis of a variety of organ systems. Thus, defining the role of sirtuins in the skin is imperative because of the potential impact it could have on dermatological care.

The skin is the largest organ in the body and consists of an outer epidermal layer that is separated from the inner dermal layer by the basement membrane. Typically, the epidermal layer consists of five layers and is constantly renewing itself *via* proliferating keratinocytes from the basal stem cell layer. As keratinocytes detach from the basement membrane, they undergo terminal differentiation which results in programmed cell death known as cornification (15). Each layer of the epidermis represents a distinct phase of this differentiation process ultimately leading to keratinocytes losing their nucleus and establishing a cytoskeleton barrier. This barrier has a variety of functions including protecting against pathogens and preventing dehydration (16–20). The inner dermal layer supports the deeper layers of the skin and consists of a large amount of extracellular matrix (15). Two distinct layers exist in the dermis—the papillary dermis and the reticular dermis. These both contain connective tissue, hair follicles, blood vessels, and sweat glands (16, 18). The papillary dermis makes contact with the basement membrane and is populated by densely packed fibroblasts, while the reticular dermis sits above the deepest layer of the skin, the hypodermis (15, 16, 18). Compared to keratinocyte populations in the epidermis, dermal fibroblasts in the dermis are rarely dividing cells that utilize damage repair mechanisms to maintain their youth (15). Dermal fibroblasts serve a variety of functions including maintaining the dermal integrity and releasing various signaling molecules that cross the basement membrane. These interact with keratinocytes to maintain skin hemostasis (18, 20, 21). Altogether, the complex interactions between the different layers of the skin help define the dermal microenvironment.

Since dermal fibroblasts are a long-lived cell type, they are vulnerable to an accumulation of damage from both intrinsic and extrinsic stressors. One consequence of these stressors is the state of cellular senescence, defined as a state of premature cell cycle arrest (15, 22). As one of the hallmarks of aging, cellular senescence can be further divided into two categories—replicative senescence and stress-induced premature senescence (SIPS) (23–25). In both cases, senescent cells can dysregulate gene expression which can lead to metabolic dysfunction and the development of the senescence-associated secretory phenotype (SASP) (26). Currently, there is not a single marker of the senescence phenotype but rather a group of characteristics a cell may express. These include a state of cell cycle arrest, macromolecular modifications, the secretory phenotype, and deregulated metabolism (27). Of interest, there will be disruption of proteins such as p16^INK4A^ and two serine/threonine kinases named Ataxia-Telangiectasia Mutated (ATM) and RAD3-related Protein (ATR), which subsequently activates p53 inhibiting the cell cycle (27). There is also a wide variety of triggers that can induce senescence such as reactive oxygen species (ROS), telomere shortening, and oncogene activation. ROS consist of (H_2_O_2_), superoxide ion (O_2_^•−^) and hydroxyl radical (^•^OH), which are by-products of oxidative metabolisms (27). Other physiological processes can become impaired as dermal fibroblast lose their ability to function properly regardless of whether they are in a state of senescence. Specifically, impaired wound healing is of concern because dermal fibroblasts are essential throughout this complex process (28, 29). Sirtuins appear to be important in the maintenance of cutaneous fibroblast activity. More precisely, it is thought that the sirtuins can impede fibroblast senescence as well as prevent the dysregulation of wound healing. As a result, the goal of this review is to establish a link between sirtuin signaling and dermal fibroblast function with a particular focus on how this information may translate to dermatologic patient care.

## 2. Fibroblast senescence

### 2.1. Background on cellular senescence

As previously stated, cellular senescence is defined as a permanent condition of cell cycle arrest and is one of the hallmarks of aging. Leonard Hayflick was one of the first to describe this process when he observed populations of cells entering a state of growth arrest after a certain number of divisions (30). Upon further investigation, a number of defining characteristics were discovered, including a number of structural changes that cause aberrant protein signaling, epigenetic modifications, and resistances to apoptotic signaling (31, 32). Additionally, biomarkers were identified, including senescence-associated-β-galactosidase (SA-β-gal), p16^INK4A^, and p53 (33). Moreover, the absence of proliferative markers, such as Ki67, can also be used to identify senescent cells (34). Senescent cells exist in a variety of different tissue types including the skin, skeletal muscle, and adipose tissue (33, 35, 36). They have also been shown to be associated with many different diseases including diabetes, hypertension, and atherosclerosis (37–39). Finally, research into this field eventually led to defining the SASP, which is a collective term applied to senescent cells that secrete excess growth factors (e.g., TGF-β), inflammatory cytokines (IL-1, IL-6, and IL-8), and matrix metalloproteinases (MMPs) (26, 40). Of interest, senescent dermal fibroblasts generate less IGF-1 which has profound effects on how the overlying keratinocyte responds to ultraviolet light and has been implicated in the increased skin cancer development of geriatric skin (reviewed in (41)).

A multitude of intrinsic and extrinsic stressors produce the accumulation of senescent cells. Specifically, *in vivo* models have been created to simulate the different stressors found in our intrinsic and extrinsic environment, such as replicative senescence and SIPS. Replicative exhaustive senescence, originally discovered by Hayflick, describes the process of telomeres shortening. Conversely, SIPS can be induced by a variety of factors, the most common being UV-induced damage and oxidative stress (23, 24, 30). Both of these models can cause excess secretion of cytokines and contribute to the development of the SASP (26). Interestingly, senescence has advantageous effects during early development and can be protective against tumor proliferation because it can prevent pre-neoplastic cells from continuously dividing (42, 43). Further, senescent cells may also help to contribute to the initial stages of wound healing by secreting platelet-derived growth factors (PDGF) (44). The accumulation of senescent cells appears to cause a shift from beneficial effects toward harmful effects. Excess and abnormal signaling leading to a state of chronic inflammation can result in tissue damage. More specifically, excess dermal fibroblast can accumulate from a variety of skin stressors, including UV damage and wound repair, all of which can disrupt skin homeostasis and contribute to the aging process (45). As senescent cells acquire the SASP, there can be deleterious effects on the skin microenvironment resulting in the secretion of proinflammatory cytokines and ultimately tumor development (46). Thus, better understanding cellular senescence and investigating potential ways to alter this response can provide integrative ways to prevent aging and other type of metabolic dysfunctions.

### 2.2. Sirtuins and stress-induced senescence

Ultraviolet (UV) light is functionally linked to skin health. As one of the many forms of light emitted from the sun, UV radiation can be further divided by wavelength into UVC (200–280 nm), UVB (280–315 nm), and UVA (320–400 nm). Since most UVC radiation is blocked by the atmosphere, it is not a concern when considering skin aging (47, 48). UVA and UVB, on the other hand, have the ability to penetrate the atmosphere and cause damage to the skin. UVA rays are absorbed largely by the dermis, where they induce the creation of ROS (49). UVB only breaches the epidermis, where it induces DNA damage by forming photoproducts such as cyclobutane pyrimidine dimers (CPDs) and 6–4 photoproducts (6-4PP) (50). The accumulation of damaged cells in the epidermis and dermis by UV light results in the upregulation of specific growth factors and cytokines, such as AP-1, which promotes the expression of MMPs (48). The accumulation of MMPs can degrade the extracellular matrix and promote the spread of tumors (48, 50, 51). Finally, ROS can stimulate the expression of NF-kB-derived proinflammatory cytokines (TNFα, IL-1, IL-6, and IL-8), all of which can magnify the UV radiation response (48). This damage can progress to photoaging, or the process of UV damage superimposed on intrinsic aging factors (52).

One consequence of photoaging is the continual disruption of cellular integrity caused by UV radiation. (53). This can lead to cellular senescence. The sirtuins have been shown to play a role in blunting this response and helping to restore photoaged skin. In general, the sirtuins prevent cellular senescence by engaging in DNA repair, preventing telomere attrition, and maintaining genome integrity (54–57). The exact process by which the sirtuins counteract UV radiation is complex and depends on the specific sirtuin family member. Moreover, the majority of the literature focuses on SIRT1, though other sirtuins have been demonstrated to play a significant role as well.

**Figure 1:**
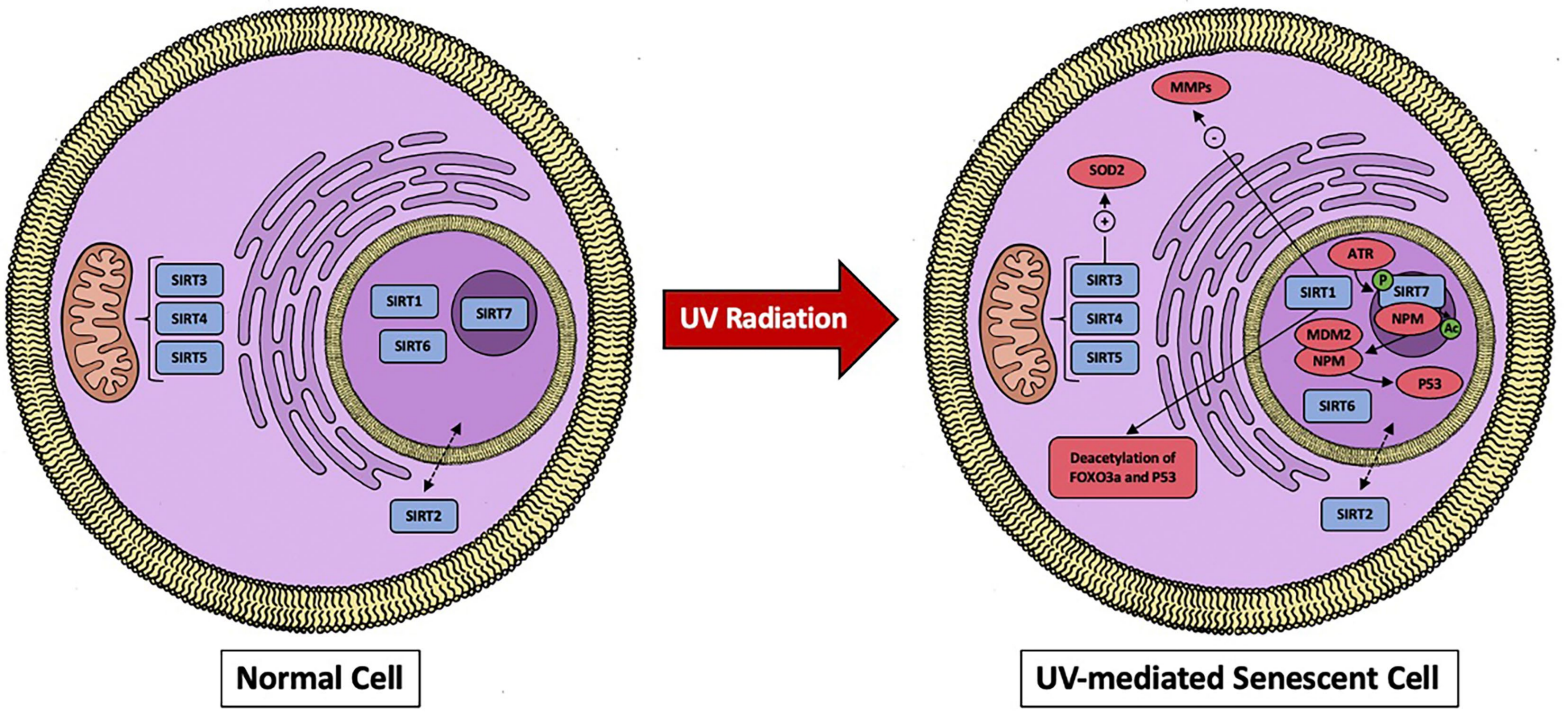
A visual representation of sirtuins localization in the cell and effects when dermal fibroblast undergoes UV-mediated damage. SIRT1 works through downregulating MMPs and by deacetylating FOXO3a and p53. SIRT7 activates p53 and the mitochondrial sirtuins (SIRT3, SIRT4, and SIRT5) will activate SOD2.

As previously mentioned, SIRT1 has the most established role in counteracting UV radiation in dermal fibroblasts. Three distinct mechanisms appear to dictate SIRT1’s ability to attenuate UV radiation; the first being through deacetylation activity of FOXO3a leading to suppression of oxidative stress (58). The second means is due to SIRT1 being found in interact with p53 to suppress UVB-induced p53 acetylation (58). Additionally, it is worth noting that excessive UV exposure has been demonstrated to lower SIRT1 levels in dermal fibroblasts, resulting in an increase in acetylated proteins. This suggest that SIRT1 activity cannot compensate for the continual damage (59). Finally, the third mechanism by which SIRT1 appears to be able to reduce UV radiation damage to dermal fibroblast is by blunting the response of MMPs. Out of the 19 MMPs produced in the skin, MMP-1, MMP-3, and MMP-9 are responsible for a majority of the UV radiation responses (Figure 1) (60). MMP-1 is a collagenase and degrades type I and III collagen. MMP-3 is a part of the stromelysins subgroup and degrades type I collagen and activates MMP-1, MMP-7, and MMP-9. Lastly, MMP-9 is a member of the gelatinases subgroup and degrades type IV collagen (61). Early *in vitro* studies done on SIRT1 by Ohguchi et al. showed that knockdown of SIRT1 by siRNA increased the expression of MMP-1 and MMP-3 in dermal fibroblast cell cultures (62). To add to this, they also found that resveratrol (a proposed SIRT1 agonist) suppressed IL-1β -mediated induction of MMP-1 (62). Further support of SIRT1’s influence on MMPs came from studies done by Lee et al., which established SIRT1’s ability to reduce the transcriptional activity of MMP-9 in dermal fibroblasts (51). Moreover, they demonstrated that SIRT1’s action was enhanced by resveratrol and metformin, leading to a reduction in the enzymatic activity of MMP-9. Interestingly, it has been recently shown that resveratrol’s activity in the cell is not through direct activation of SIRT1, but rather the drug’s induction of low-level replicative stress on cell proliferation that can upregulate SIRT1 (63). *In vivo* studies have also shown SIRT1’s involvement in UV-induced damage. More specifically, SIRT1 expression was found to be low in normal skin. However, it is significantly increased after both low and high doses of UVA radiation, suggesting a protective mechanism in preventing UV damage (64). As a result, direct activators of SIRT1, as well as innovative ways to alter SIRT1 expression, could be useful in reducing UV damage, thereby preventing the accumulation of senescent cells.

Enhancing SIRT1’s ability to augment UV damage represents one strategy to prevent cellular senescence and restore fibroblast function. Several *in vitro* SIRT1 activators that work through the methods described above have been described (59, 65–71). For example, in UVA-induced senescent dermal fibroblasts, pyrroloquinoline quinine (PQQ), a redox co-factor isolated from methylotrophic bacteria, recovered the protein expression of SIRT1, as well as SIRT6. Further, it reduced the activity of MMP1 and MMP3 compared to the non-treated control (66). Moreover, Aquatide, a synthetic SIRT1 activator, was found to accelerate autophagy induction and reduce UVB-induced cellular senescence (71). Additionally, shikimic acid has also been identified to act indirectly on the SIRT1 pathway and prevent UV-induced cellular senescence in dermal fibroblasts (69). Red light irradiation has also been demonstrated to restore sirtuin function. More specifically, Niu et al. exposed dermal fibroblast to UVA and UVA plus red light. Red light irradiation also increased SIRT1 expression, and protected dermal fibroblasts from UVA-induced senescence (72). Recently, *in vivo* models were used to test the effects of a 50% ethanol extract from *Nypa fruticans* (NF50E), a plant belonging to the family of *Aceraceae* (67). SIRT1 secretion in the dermis was reduced in a UVB-induced animal model, where topical administration of NF50E enhanced SIRT1 expression, repressed the activation of AP-1 and NF-kB, and constrained collagen degradation by MMP-1 (67). Finally, Galangin, a flavonoid found in *Alpinia Officinarum*, has also been found to protect against UVB-induced skin photoaging in nude mice *via* the upregulation of SIRT1 and subsequent activation of heme oxygenase-1 (HO-1), an antioxidant enzyme (73).

The involvement of the remaining sirtuins in UV protection and cellular aging is still being investigated. SIRT3 has been found to protect against UV-induced senescence *in vitro* and *in vivo via* an A2AR/SIRT3/AMPK-mediated autophagy pathway and through enhanced superoxide dismutase 2 (SOD2) activity. All of these can lead to enhancements in the ability of mitochondria to diminish oxidative stress [66, 67, (74)]. Interestingly, caffeine has direct SIRT3-activating effects and has been shown to prevent collagen degradation in UV-irradiated mouse skin (75). Moreover, the expression of SIRT4 is increased in dermal fibroblast undergoing UVB-induced senescence as well as replicative senescence *in vitro* (76). Additionally, SIRT4 appears to be regulated by micoRNA-15b, which can prevent this stress-induced increase in SIRT4 expression (76). MicroRNA expression has an effect on SIRT6 expression as well. Specifically, microRNA-378b is induced in UVB-exposed human dermal fibroblasts. This subsequently represses the mRNA expression levels of a-1-type 1 collagen (COL1A1) by disrupting SIRT6 activity (77, 78). Arctin, a lignin derived from *Arctium lappa*, downregulates microRNA378b, preventing UVB-induced reduction in COL1A1 and restoring SIRT6 activity (78). Recently, *in vitro* studies by Lee et al. revealed that UV-irradiated dermal fibroblasts had lowered SIRT2 and SIRT3 mRNA and protein levels (79). Surprisingly, there was no discernible decrease in the remaining sirtuins. Overexpression of HDAC4, a class II histone deacetylase, has also been shown to prevent cellular senescence (79). Finally, Lanni et al. discovered that SIRT7 is crucial for maintaining the integrity of the tumor suppressor p53 during UV radiation. More specifically, they demonstrated UV irradiation enhances SIRT7 activity through ataxia telangiectasia mutated and Rad3 related (ATR)-mediated SIRT7 phosphorylation, resulting in deacetylation of nucleophosmin (NPM) which binds MDM2, preventing MDM2-mediadted proteasomal degradation of p53, causing an enhanced activation of p53. (80). In conclusion, each sirtuins role in UV-induced damage is still being established, as are the molecular processes that underlie them. However, it is necessary to investigate real-world applications with a stronger emphasis on *in vivo* models and therapeutic possibilities in humans.

The literature on sirtuin functions in an oxidative stress model and replicative stress model of dermal fibroblast senescence is limited but worthy of discussion. The production of oxidative stress is due to a delicate balance between ROS and detoxifying strategies. As discussed previously, different types of ROS exist including superoxide radicals (O_2_^•−^), hydrogen peroxide (H_2_O_2_), hydroxyl radicals (•OH), and singlet oxygen (^1^O_2_). These are all produced as metabolic by-products from various sources including mitochondrial electron transport chain, the peroxisome, and UV-mediated accumulation (81, 82). Defense mechanisms, including superoxide dismutase and catalases, are in place. However, when the defensive capacity of these mechanisms is exceeded, alteration occur including mitochondrial phospholipid peroxidation, cellular signaling dysregulation, protein transport malfunction, and peroxisomal dysfunction. Cellular senescence may then be initiated in order to prevent the proliferation of abnormal cells (81, 83, 84). To further elaborate on how oxidative stress specifically causes senescence, Zhu et al. have summarized four molecular pathways that are contributing the dysfunction discussed above. The first being oxidative stress causes DNA damage activating the DNA damage response pathway through activating of p53 and upregulating p21. The second being due to oxidative stress phosphorylating IκB causing activation NF-κB and stimulation of IL-8. The third due to p38 MAPKs pathway in which activated ROS upregulate p19 protein expression and the fourth the influence of oxidative stress on microRNA function and the promotion of cellular senescence (84).

**Figure 2:**
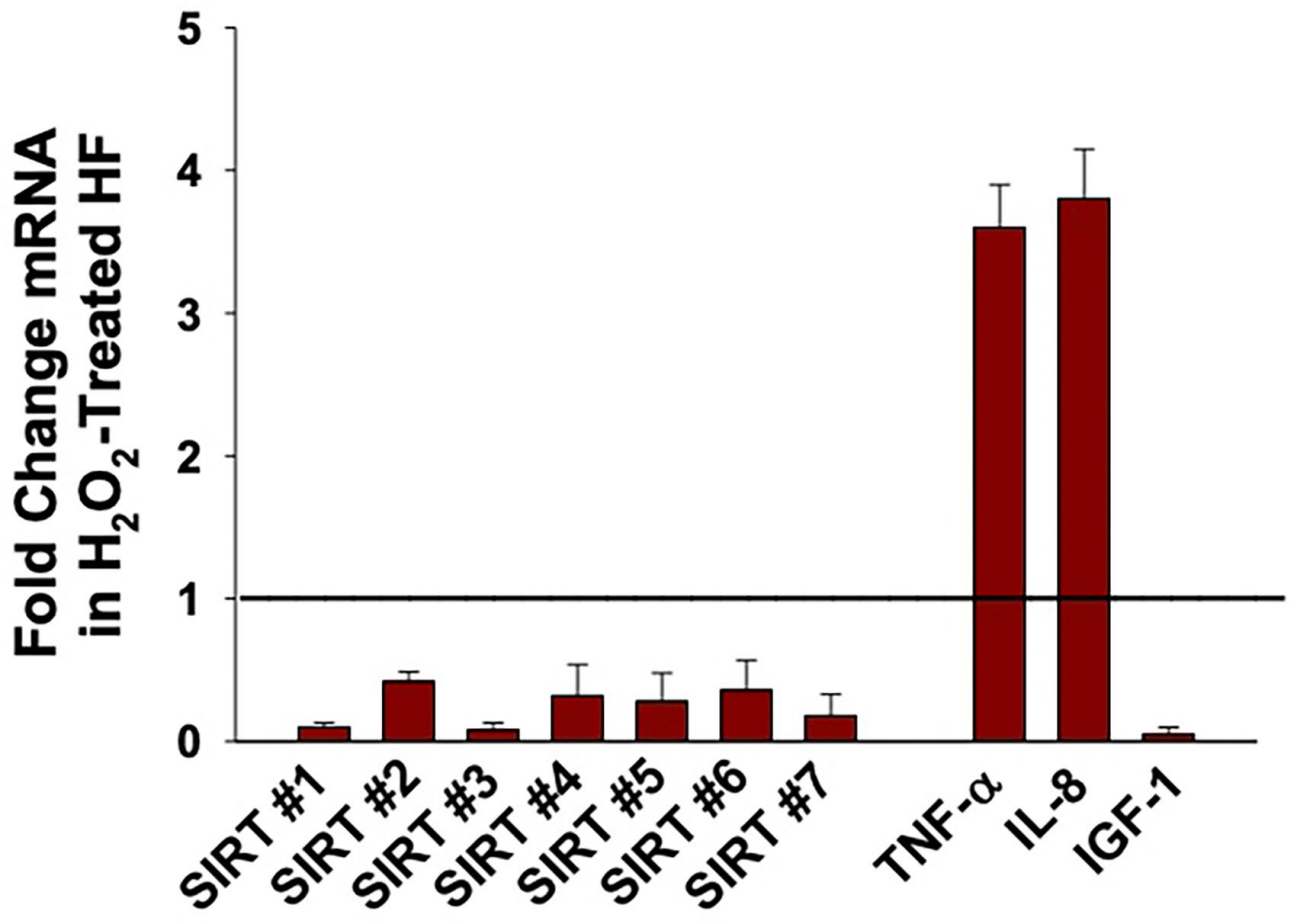
Effect of Hydrogen Peroxide (H_2_O_2_) on the expression of Sirtuins (1 to 7), TNFα, IL-8, and IGF-1 in oxidant-induced senescent Dermal Human Fibroblasts (DHF). Neonatal-derived HF were treated with 600 μM of H_2_O_2_ for 2 h, for oxidant-induced cell senescence and then harvested 72 h later. The data are mean ± SE levels of mRNA from different sirtuins (1–7) normalized to 18 s and cytokines relevant to fibroblast senescence from 3 to 4 different experiments using triplicate samples. The statistical analysis was done using Student t test where all values listed are statistically (*p* < 0.05) different from control values set at 1 (see line). Please see reference (87) for further details as to the characterization of the DHF treated with vehicle vs. pro-oxidative stressor.

Early *in vitro* studies on oxidative stress and SIRT1 demonstrated that expression of SIRT1 is diminished when dermal fibroblasts are treated with H_2_O_2_ (85, 86). To add to the limited literature on H_2_O_2_-induced fibroblast senescence, our group treated senescent fibroblasts with 600 μM H_2_O_2_ and measured the mRNA expression of all seven sirtuins (Figure 2). Our previously published study describes the process of oxidant induced cell senescence in detail including the dose response curve for H_2_O_2_ and the method by which the mRNA was isolated, as well as the finding that the oxidant-treated fibroblasts have increased levels of p21, and SASP-associated cytokines IL-6, IL-8, and TNFα (87). Our findings reveal that the expression of all seven sirtuins were significantly reduced following treatment with H_2_O_2_ (Figure 2). Thus, the low levels of sirtuins may not be able to mitigate the potential damage to the senescent cells caused by excess oxidative damage. Relevant to the role of potential fibroblast senescence in non-melanoma skin cancer, oxidant-treated fibroblasts also had diminished levels of insulin-like growth factor 1 (IGF-1) (41, 87). More specifically, dermal fibroblast produce IGF-1 that acts in a paracrine fashion on IGF-1 receptors (IGF-1R) expressed on keratinocytes. This activates a variety of different pathways responsible for cell proliferation and apoptosis (88). When fibroblasts undergo senescence, IGF-1 secretion is impaired, thereby causing keratinocyte dysfunction and increasing the susceptibility of potential for non-melanoma carcinogenesis (89). Interestingly, our previously published data demonstrated pretreatment with creatine and nicotinamide (NAM) protects dermal fibroblasts from H_2_O_2_-induced cell senescence (87). Moreover, NAM is rapidly converted to NAD^+^ in cells through sirtuin deacetylation activity, suggesting that IGF-1 and sirtuin regulation may influence each other through its enzymatic reactions including NAM (87, 90, 91). The potential relationship is further supported by other literature showing sirtuins can inhibit IGF-binding proteins, which can inhibit IGF-1 function (92). However, relationship between IGF-1 and the sirtuins remains unclear because high NAM levels may actually inhibit SIRT1 through a negative feedback mechanism (93).

More recently, a multitude of *in vitro* studies have focused on preventing the downregulation of SIRT1 in H_2_O_2_-mediated dermal fibroblast cellular senescence, either directly or indirectly through a variety of different compounds (73, 94–98). Letsiou et al. treated H_2_O_2_-damaged dermal fibroblast with an *Aspergillus chevalieri* extract and found that it was capable of restoring the levels of SIRT1 and SIRT2 (98). Another study looked at the effects of pollutant particulate matter on dermal fibroblasts, which was found to cause oxidative stress, cellular senescence, as well as reduced SIRT1 expression in a concentration-dependent manner (99). Moreover, the literature on replicative fibroblast senescence is considerably limited, yet there are a few intriguing papers out there. Fridman and investigators reported that AED peptide triggered the synthesis of both SIRT1 and SIRT6 in dermal fibroblast undergoing replicative aging (100). Finally, SIRT4 mRNA levels were significantly increased during replicative senescence in human foreskin fibroblasts (76). Thus, it is unclear what the mechanisms is behind increased levels of SIRT4 mRNA levels in a replicative stress model when compared with our oxidant-induced senescence model. Importantly, our studies demonstrate that the levels of sirtuin 1–7 expression were significantly decreased in H_2_O_2_- treated oxidant-induced senescent dermal human fibroblasts (DHF) compared to vehicle control-treated DHF (Figure 2), indicating that conditions resulting in oxidative stress can downregulate sirtuins.

## 3. Wound healing

### 3.1. Physiological response to dermal injury

Wound healing involves a set of complex interactions between different cell types and signaling molecules that ultimately leads to the restoration of the skin’s barrier. Typically, wound healing has been described in three distinct phases: the inflammatory, proliferation, and maturation and remodeling phases (101). The inflammatory phase encompasses hemostasis and inflammation, which leads to the activation of the coagulation cascade, and migration of neutrophils and macrophages (101, 102). The proliferative phase produces the formation of granulation tissue, re-epithelization, angiogenesis, and the production of collagen to build a new extracellular matrix (101–103). Finally, the maturation and remodeling phase strengthens the wound with deposition of additional collagen in an organized manner (101). Keratinocytes, endothelial cells, immune cells, and dermal fibroblast interact with each other through this process *via* various cytokines to enable healing.

Dermal fibroblasts are multifactorial in wound healing because of their location within the dermis. Fibroblasts will migrate into the wound to form granulation tissue and deposit collagen for the new extracellular matrix (103). Some of these fibroblasts will be induced by transforming growth factor beta (TGFβ) to become myofibroblasts, which will assist in wound contraction (104). The interactions between dermal fibroblasts and other cell types in the skin during wound healing can be described in two distinct ways. First, dermal fibroblasts will interact with immune cells and keratinocytes directly in the inflammatory and proliferative phases that mediate cell migration, proliferation and adhesion. Second, dermal fibroblasts will undergo paracrine interactions between immune cells and keratinocytes during the inflammatory and proliferative phase. This will lead to inflammatory and anti-inflammatory effects through M1 and M2 macrophages and enhance the re-epithelization process, MMP-1 synthesis, and collagen synthesis (102).

Ultimately, there is potential for this process to malfunction, leading to two potential outcomes: chronic wound healing and excessive scar formation. Fibroblasts can become unresponsive during wound healing, leading to an upregulation of growth factors and MMPs, as well as a dysregulation of the TGFβ signaling pathway (105, 106). The second outcome involves excessive formation of scar tissue, which can develop into a hypertrophic or keloid scar. The literature on the wound healing process is extensive. Nevertheless, it is important to continue to investigate this process in order to recognize potential targets, enhance acute wound repair, and prevent chronic complications.

### 3.2. Sirtuins relationship with wound healing

**Figure 3:**
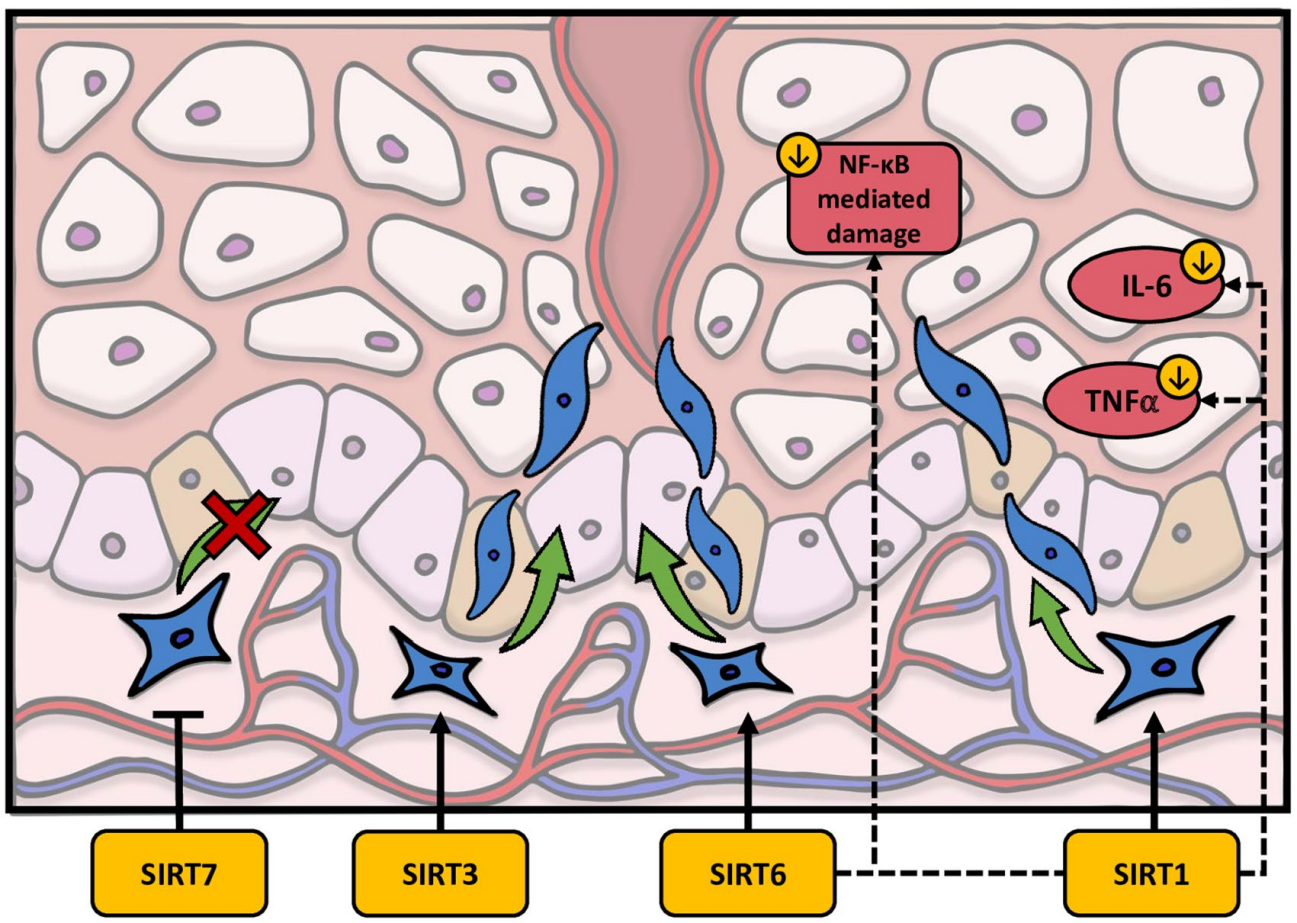
The effects of sirtuins on the dermal wound healing process. SIRT1 downregulates IL-6 and TNFα as well as activating fibroblast migration. SIRT3 and SIRT6 have also been shown to promote fibroblast migration. Finally, SIRT7 may inhibit fibroblast migration.

In terms of fibroblast function, sirtuins have been found to play a variety of roles in wound healing. As with fibroblast senescence, SIRT1 has the most established role in wound healing. In particular, Qiang et al. showed that fibroblast recruitment and activation is inhibited in epidermis-specific deletion of SIRT1 knockout (KO) mice (107). Wildtype (WT) mice had greater activity of α-SMA^+^ cells, a marker of activated fibroblast, in granulation tissue compared to the KO mice (107). Interestingly, SIRT1 may also play a role in hypertrophic scars. In a mouse model of wound healing, Bai et al. observed that SIRT1 deletion causes more disorder within the skin with denser collagen fibers, whereas resveratrol treatment improved these processes (108). To support these findings, Bai et al. also discovered that the depletion of SIRT1 showed an upregulation of α-SMA^+^, Col11, and Col3 in hypertrophic scar-derived human dermal fibroblasts, suggesting it may have a role in maintaining the balance of certain molecules in wound repair (108). Furthermore, increased levels of SIRT1 also prevented TGFβ-induced activation of normal dermal fibroblasts. (108). SIRT1 has also been linked to diabetic wound healing due to its ability to inhibit NF-κB-mediated tissue damage (109, 110). More specifically, SIRT1 downregulation can contribute to a proinflammatory state through an increase in the NF-κB-mediated pathway, which upregulates TNFα and IL-6 (Figure 3) (109). Recently, however, activators of SIRT1 have been investigated to improve the process of wound healing. Berberine, an alkaloid extract from *philodendron amurense*, has been shown in a diabetic rat model to work by activating SIRT1 and inhibiting the expression of NF-κB mediated, TNFα, and IL-6 (109). NED416 is another SIRT1 activator demonstrated to promote SIRT1 activity in dermal fibroblast and epidermal keratinocytes, as well as accelerate wound closure and collagen formation both *in vitro* and *in vivo* (111). Furthermore, it appears that NED416 is more potent than resveratrol and enhances cell migration *via* the Rac1/Cdc42 pathway and MAPK signaling (111). Finally, utilizing a rat model, a combination of caloric restriction and resveratrol have been suggested to increase SIRT1 activation and regulate angiogenesis, fibroblast proliferation, and collagen production (112).

The remaining literature concerning the role of sirtuins in wound healing pertaining to dermal fibroblasts is limited but is worthy of discussion. Recent work by Yang et al. demonstrated that SIRT3 expression is decreased in the skin of diabetic patients (113). This group expanded upon this finding by determining that SIRT3 deficient diabetic mice have diminished skin fibroblast migration as well as reduced blood supply and delayed healing rates (113). Inasmuch SIRT3 is a mitochondrial sirtuin, Yang et al. hypothesized that the delay in wound repair caused by SIRT3 loss could be related to mitochondrial malfunction, increased oxidative stress, or amplified necroptosis (113). Interestingly, SIRT6 may work in a similar manner as SIRT1 in wound repair, but the exact mechanism on dermal fibroblasts in wound repair remains unclear. The inhibition of SIRT6 may promote a proinflammatory response by increasing NF-κB and decreasing angiogenesis in diabetic mice. Yet, more research is needed to define its role in dermal fibroblasts (114). In recent years, SIRT7 has also been identified for its potential role in wound healing (115, 116). Noteworthy of discussion, Xia et al. found that exosomal transfer of micro-RNA-125b (miR-125b) inhibits SIRT7 in fibroblasts in a wound healing mice model, which actually increases myofibroblast differentiation in aged fibroblasts (116). They also discovered that, as the amount of SIRT7 decreases, the number of α-SMA^+^ fibroblasts increase, thus contributing to the evidence that SIRT7 activation inhibits old fibroblasts and prevents fibroblast migration (116). This may propagate potential interest in discovering new outlets for promoting wound healing. In sum, the literature surrounding sirtuins and wound healing suggest they may have a protective role through a variety of mechanisms including fibroblast migration, inhibition of NF-κB-mediated tissue damage, and regulation of angiogenesis. However, a contradiction exists in the literature. As discussed previously Demaria and colleagues demonstrated senescent fibroblasts arise very early during the healing of wounds and encourage wound closure by stimulating myofibroblast differentiation through the release of PDGFs (44). This discrepancy may be explained by the authors noting that senescent fibroblasts are present only transiently during tissue repair (44). Thus, it possible that sirtuins potentially protective role is based on timing and cellular dynamics within the wound healing process.

### 3.3. Human disease models and clinical applications

The role of fibroblasts and sirtuins in certain diseases and cosmetic applications is being currently examined (117–125). Most notably, systemic sclerosis (SSc) has been investigated in regards to sirtuin activity, which is an immune-mediated rheumatic disease that causes fibrosis of the skin and other organs (126). SIRT1 mRNA in SSc is diminished in both mouse models and human skin biopsy samples (117, 118). However, the exact pathogenesis remains unclear because different studies have showed opposite results. Wei et al. showed that the pro-fibrotic effect of TGFβ was diminished by activation of SIRT1, while Zerr et al. showed downregulate of SIRT1 diminished TGFβ/Smad signaling (117, 118). More recently, Manetti et al. measured circulating SIRT1 and SIRT3 levels in a cohort of SSc patients and healthy controls, and found significantly decreased serum levels of both SIRT1 and SIRT3, as well as an association with the extent of skin involvement (127). Further, Akamata et al. revealed that SIRT3 activity was significantly reduced in SSc biopsies and that its activation can augment TGFβ signaling and the fibrotic response (123). Another disease for which sirtuins have been implicated as a therapeutic target is in mitochondrial cytochrome c-oxidase (COX or CcO) deficiency. Potthast et al. examined SIRT1, SIRT3, and SIRT4 in human skin fibroblasts from COX deficient patients and found significantly decreased levels of all three (121). Additionally, they were able to modulate sirtuin levels and alter respiratory chain function through the sirtuin activator, SRT1720, and paeonol (121). As stated previously, the discovery of sirtuins occurred in the Saccharomyces cerevisiae yeast, which is the yeast that patients with inflammatory bowel disease can make antibodies to called Anti-Saccharomyces cerevisiae antibodies (ASCAs). Thus, sirtuins may influence inflammatory bowel diseases including Crohn’s disease and Ulcerative Colitis (128, 129). There may be a connection between sirtuin dysregulation and extra-intestinal skin issues including erythema nodosum and pyoderma gangrenous, although there is currently no formal research on the subject.

Recent studies have provided compelling evidence linking the aging of dermal fibroblasts, with corresponding lack of IGF-1, as playing an important role in the increased incidence of non-melanoma skin cancer (NMSC) in the elderly (41, 89). Our current data indicating that the mRNA levels of all seven sirtuins were diminished in senescent normal human fibroblast suggest that these bioactive agents could be playing a role in the fibroblasts SASP. Uncertainty exists within the present literature on the role of sirtuins in NMSC. All seven sirtuin mRNA levels were overexpressed in one study involving squamous cell cancer cell lines and actinic keratosis (130). Another study on squamous cell carcinoma (SCC) found lower levels of SIRT2 in tumors and that SIRT2 deletion enhances the risk of carcinogenesis (131). Additionally, SIRT6 may play a pro-proliferative role in basal cell carcinoma (BCC) and SCC (132). IGF-1 levels are lower in SIRT6 KO mice, indicating that SIRT6 may be crucial for IGF-1 regulation (133). Future studies should seek to determine whether the lack of sirtuins in the dermal NHF are playing a passive or active role in age-associated NMSC. There is still more to learn about how sirtuins and fibroblast function relate to other kinds of skin cancer, like dermatofibrosarcoma protuberans (DFSP). DFSP is a cutaneous sarcoma derived from fibroblast and multiple studies have linked its function to EZH2-mediated histone methylation, and COL1A1-PDGFB translocation (134, 135). As mentioned previously, SIRT6 is influenced through mRNA express of COL1A1 activity (77, 78). There is no formal literature on this subject but SIRT6 may be contributing to the pathogenesis of DFSP through COL1A1 mediated activity.

The sirtuins make up the histone class III deacetylates. Therefore, HDAC inhibitors have the potential to alter the sirtuins activity and alleviate different processes in the diseases described above. For example, HDAC inhibitors TSA and Divalproex Sodium have both been shown to be used to treat systemic sclerosis in mouse models and humans (136–139). Additionally, Cutaneous T-cell Lymphomas (CTCL) are also treated with HDAC class I and II inhibitors including Vorinostat and Romidepsin. This heterogeneous group of diseases consist of a monoclonal proliferation of T-lymphocytes and primarily involve the skin. They commonly cause fibrosis in the skin through and IL-4 and IL-13 interaction with dermal fibroblast (140, 141). SIRT1 was found to be overexpressed in cell lines of CTCL (142). Tenovin-1, a class III HDAC inhibitor of SIRT1 and SIRT2, reduces SIRT enzymatic activity, causing apoptosis (142). Finally, although not the focus of this paper, it is worthy of mention that HDAC inhibitors are also indicated in treating melanoma in which they can induce apoptosis by downregulating sirtuin activity in cancer cells (143–145).

Recently, there have been multiple publications related to other skin pathologies in which sirtuin alteration may have clinically relevant dermatological applications (146–152). For example, resveratrol and its analogs resveratryl triacetate (RTA) and resveratryl triglycolate (RTG) have been tested in multiple clinical studies. Studies testing 1% resveratrol cream and a combination of different creams of RTA and RTG have been tested in which 0.8% RTA showed antiaging effects by improving various skin parameters such as skin wrinkles, and elasticity (120, 153, 154). Sirtuin activation could be playing a role in this process but it is currently unclear as resveratrol can work through multiple different mechanisms (120). Rosacea is a chronic inflammatory disease that presents with recurrent flushing, erythema, telangiectasia on the nose, cheeks, and forehead (155). Interestingly, loss of SIRT7 alleviates the rosacea-like features in mice models and human dermal fibroblast. More specifically, epidermal keratinocytes in rosacea inflamed skin activates toll like receptors (TLR2) leading to the release of various cytokines. SIRT7 is upregulated in skin samples of patients and in mouse models with rosacea and can regulate gene transcription of TLR2 which activates NF-kB. Thus, this SIRT7-TLR2- NF-kB pathway may be one mediator of the inflammatory process in rosacea (146).

Other pathologies that are not directly derived from fibroblast dysfunction are also worthy of discussion due to the fluidity of the dermal microenvironment. Systemic lupus erythematous (SLE), an autoimmune inflammatory condition with an unclear pathophysiology which exhibits a variety of skin manifestation including a malar rash, discoid rash, photosensitivity, and oral mucosal lesions (156). A recent review analyzed SIRT1 and SLE and found that resveratrol was able to alleviate inflammation and decrease the levels of autoimmune antibodies. However, Qiu and colleagues also noted SIRT1 levels may be upregulated in CD4^+^ cells and they reported there is currently a lack of clinical evidence to recommend using SIRT1 activators in humans (147). Psoriasis is another chronic inflammatory disease mediated through the activation of T cells which stimulate the proliferation of keratinocyte. Applications of sirtuins in psoriasis have been extensively studied (149, 151, 157–160). In a recent study D’Amico and colleagues, took punch biopsies from 6 untreated female patients affected with chronic plaque psoriasis. Immunohistochemical analysis was done and reduction of SIRT1 and adenosine monophosphate-activated kinase (AMPK) expression was observed (158). Vacharanukrauh and colleagues analyzed 9 patients with psoriasis treated with before and after UVB-mediated phototherapy. They concluded UVB-mediated phototherapy works partially through SIRT1 anti-inflammatory effects downregulating NF-kB. All patients showed clinical improvement their lesions after therapy (161). As mentioned previously NAM is rapidly converted to NAD^+^ in cells through sirtuin deacetylation activity. It has been shown to be beneficial in a variety of skin diseases partially through SIRT1-mediated deacetylation downregulated pro-inflammatory transcription of NF-kB. Oral NAM shown efficacy both in oral and topical applications in bullous pemphigoid, acne vulgaris, and rosacea as well as for the treatment and prevention of NMSC in a variety of clinical trials (162–167). It is indeed possible that NAM effects are *via* SIRT1. In sum, there are several opportunities to focus on methods of treating diseases that may directly or indirectly influence the dermal fibroblast or other cells in the dermal microenvironment. Sirtuin alteration by oral and topically applied treatments are still in the early stages but there is growing accumulation of human data through clinical trials.

## 4. Conclusion

Investigating the function of sirtuins within the dermal microenvironment has garnered more attention recently. The sirtuins seem to be crucial for both fibroblast senescence and wound healing. As a result, this review explores how sirtuins affect dermal fibroblast function and provide additional experimental evidence that indicates that expression levels of all sirtuins are decreased in the H_2_O_2_-induced fibroblast senescent cells. We also looked at the function of sirtuins in diseases like systemic sclerosis and how sirtuins may relate to NMSC and IGF-1. In summary, the body of research investigating sirtuins and fibroblast function in specific disease states is still in its infancy. However, continued study may shed light on clinically relevant opportunities where the alteration of sirtuin function may provide benefits to certain patient populations.

## Author contributions

MG, CR, JT, and AT: conceptualization. MG, SM, AM, and AT: methodology. MG, AM, CR, JT, and AT: validation. MG, AM, and AT: investigation. CR, JT, and AT: resources. MG: writing—original draft preparation. SM, CR, JT, and AT: writing—review and editing. CR, JT, and AT: supervision and project administration. JT: funding acquisition. All authors contributed to the article and approved the submitted version.

## Funding

This work was supported by grants from the National Institutes of Health (R01 HL062996 to JT and R01 AG048946 to JT), VA Merit Award (1101CX000809 to JT). The content is solely the responsibility of the authors and does not necessarily represent the official views of the National Institutes of Health or the US Veterans Administration.

## Conflict of interest

The authors declare that the research was conducted in the absence of any commercial or financial relationships that could be construed as a potential conflict of interest.

## Publisher’s note

All claims expressed in this article are solely those of the authors and do not necessarily represent those of their affiliated organizations, or those of the publisher, the editors and the reviewers. Any product that may be evaluated in this article, or claim that may be made by its manufacturer, is not guaranteed or endorsed by the publisher.
